# Circulating LOXL2 Levels Reflect Severity of Intestinal Fibrosis and GALT CD4^+^ T Lymphocyte Depletion in Treated HIV Infection

**DOI:** 10.20411/pai.v2i2.180

**Published:** 2017-06-21

**Authors:** Sophie Seang, Anoma Somasunderam, Maitreyee Nigalye, Ma Somsouk, Timoty W. Schacker, Joyce L. Sanchez, Peter W. Hunt, Netanya S. Utay, Jordan E. Lake

**Affiliations:** 1 Pitie Salpetrière Hospital, University Pierre et Marie Curie, Paris, FRANCE and INSERM UMR-S943; 2 University of Texas Medical Branch, Galveston, Texas; 3 University of California, San Francisco, California; 4 University of Minnesota, Minneapolis, Minnesota; 5 Mayo Clinic, Rochester, Minnesota; 6 University of Texas Health Science Center at Houston, Houston, Texas

**Keywords:** GALT, HIV, fibrosis, immune reconstitution

## Abstract

**Background:**

Incomplete immune reconstitution may occur despite successful antiretroviral therapy (ART). Gut-associated lymphoid tissue (GALT) fibrosis may contribute via local CD4^+^ T lymphocyte depletion, intestinal barrier disruption, microbial translocation, and immune activation.

**Methods:**

In a cross-sectional analysis, we measured circulating fibrosis biomarker levels on cryopreserved plasma from adult HIV-infected (HIV+) SCOPE study participants on suppressive ART who also had fibrosis quantification on recto-sigmoid biopsies. Relationships among biomarker levels, clinical and demographic variables, GALT lymphoid aggregate (LA) collagen deposition, and LA CD4^+^ T lymphocyte density were analyzed using simple regression. Biomarker levels were also compared to levels in HIV+ viremic SCOPE participants and a convenience sample of HIV-uninfected (HIV-) samples.

**Results:**

HIV+ aviremic participants (n = 39) were 92% male and 41% non-white, with median age 48 years, CD4^+^ T lymphocyte count 277 cells/mm^3^, and 17 years since HIV diagnosis. Most biomarkers were lower in HIV− (n = 36) vs HIV+ aviremic individuals, although CXCL4 levels were higher. HIV+ viremic individuals (N = 18) had higher median TGF-ß_3_, CIC-C1Q, and TIMP-1 (*P* < 0.05) and lower LOXL2 levels (*P* = 0.08) than HIV+ aviremic individuals. Only higher LOXL2 levels correlated with more GALT collagen deposition (R = 0.44, *P* = 0.008) and lower LA CD4^+^ T lymphocyte density (R = −0.32, *P* = 0.05) among aviremic individuals.

**Conclusions:**

Circulating LOXL2 levels may be a noninvasive measure of intestinal fibrosis and GALT CD4^+^ T lymphocyte depletion in treated HIV infection. LOXL2 crosslinks elastin and collagen, and elevated LOXL2 levels occur in pathologic states, making LOXL2 inhibition a potential interventional target for intestinal fibrosis and its sequelae.

## INTRODUCTION

Despite control of HIV replication with antiretroviral therapy (ART), long-term failure to restore CD4^+^ T lymphocyte numbers has been reported in HIV-infected (HIV+) individuals [1, 2] Additionally, poorer immune reconstitution has been associated with the development of non-AIDS comorbidities and decreased lifespan among HIV+ persons [3]. The pathophysiology of failure to achieve immunologic recovery is not fully understood; however, collagen deposition and fibrosis in lymphoid tissues, as observed in gut-associated lymphoid tissue (GALT) during HIV infection, may contribute to incomplete immune reconstitution by limiting GALT plasticity and immune cell homeostasis [4, 5].

Fibrosis occurs when the normal healing response to local inflammation becomes perturbed, resulting in an imbalance between deposition and degradation of the extracellular matrix (ECM) [6]. Fibroblasts in tissues produce ECM components such as collagen and hyaluronic acid (HA). Anti-fibrotic and pro-fibrotic mediators such as chemokines, cytokines, and growth factors (especially transforming growth factor [TGF]-β_1_) [7] direct tissue remodeling. ECM turnover also involves the innate and adaptive immune systems through production of inflammatory mediators [8]. Over time and in the face of a persistent inflammatory stimulus such as HIV infection, progressive fibrosis occurs that may ultimately compromise organ function.

In this study, we analyzed the relationships between GALT collagen deposition, intestinal lymphoid aggregate (LA) CD4^+^ T lymphocyte density, clinical/demographic variables and mediators of fibrosis (TGF-ß_1_-_3_), matrix metalloproteinase (MMP)-2 and -9, tissue inhibitor of MMP (TIMP)-1, chitinase-3-like protein 1 (CHI-3L1), HA, lysyl oxidase-like 2 (LOXL2), [9, 10] type I C-terminal collagen pro-peptide (CICP), circulating immune complexes of complement 1q (CIC C1Q), [11] plasminogen activator inhibitor-1 (PAI-1), CXC chemokine ligand 4 (CXCL4), [12] and pro-protein convertase subtilisin/kexin type 9 (PCSK9). We hypothesized that circulating levels of these fibrosis biomarkers would be associated with the severity of GALT collagen deposition and LA CD4^+^ T lymphocyte depletion in ART-treated, HIV-1-infected adults.

## PATIENTS AND METHODS

### Study Population

We utilized data and plasma samples from participants enrolled in the Observational Study of the Consequences of the Protease Inhibitor Era (SCOPE), a longitudinal cohort of HIV+ and HIV-uninfected adults followed in San Francisco, California. A subset of 73 SCOPE participants underwent recto-sigmoid biopsies and had stored plasma available for biomarker assessment. For this cross-sectional analysis, the primary population was further restricted to the subset of participants (n = 39) with HIV-1 RNA < 50 copies/mL. As secondary endpoints, a group of viremic (HIV-1 RNA > 50 copies/mL) HIV+ individuals (n = 18) from the SCOPE study and a convenience sample of unmatched HIV-uninfected (HIV−) individuals (n = 36) were used for comparison analysis of biomarker values.

The SCOPE cohort was approved by the University of California-San Francisco (UCSF) Committee on Human Research. All subjects provided informed consent prior to enrollment, and the human experimentation guidelines of UCSF were followed in the conduct of clinical research.

### Specimen collection and analysis

Rectal biopsy specimens were stained with Masson trichrome to identify collagen fibers and with CD4 antibody, as previously described [13]. Collagen abundance and the CD4^+^ T lymphocyte population were assessed using quantitative image analysis.

### Clinical parameters

Clinical and demographic data and medical and medication history were extracted from the SCOPE database.

### Circulating fibrosis biomarkers

Levels of fibrosis biomarkers were measured on EDTA plasma by ELISA or multiplex assay using commercially available kits. HA (Echelon Biosciences, Salt Lake City, UT), LOXL2 (R&D Systems, Minneapolis, Minnesota), and CICP and CIC C1Q (Quidel, San Diego, CA) were measured by ELISA. PAI-1, PCSK9, TGF-ß_1_-_3_, MMP-2, MMP-9, TIMP-1, CXCL4, Chi3L1 (all R&D Systems, Minneapolis, MN) were measured using Luminex xMAP technology. All analytes except LOXL2 were measured according to the manufacturers' instructions. For LOXL2, the ELISA plates were coated overnight with capture antibody at room temperature. The plate was blocked with reagent diluent 1 (R&D Systems, Minneapolis, MN) for 1 hour and washed. Plasma was diluted 1:10 in PBS-Tween 10 and added to the plate. After incubating for 2 hours at 37° C and washing, detection antibody was added for 2 hours at 37° C. The plate was washed, streptavidin was added and the plate was incubated for 20 minutes at room temperature. After washing, TMB was added (Sigma-Aldrich Corp., St. Louis, MO), the plate was incubated for 20 minutes at room temperature, and sulfuric acid was added to stop the reaction. The plate was read at 450 nm.

### Statistical methods

Continuous variables are presented as medians and interquartile ranges (IQR), and nominal data are described as absolute values or percentages. Statistical analyses were performed using the Mann-Whitney U-test. Associations were assessed using simple regression and Spearman correlation coefficients. Significance was defined as two-sided α≤0.05. All analyses were exploratory, without adjustment for multiple testing.

## RESULTS

### Study population

Among the 39 ART-treated, aviremic HIV+ individuals with recto-sigmoid biopsy data and available plasma for biomarker analysis, 92% were male. Median (IQR) age was 48 (45-55) years, nadir CD4^+^ T lymphocyte count cells was 66 (18-108) cells/mm^3^, and current CD4^+^ T lymphocyte cell count was 277 (177-483) cells/mm^3^ (Table 1). Characteristics of viremic HIV+ individuals (n = 18, 9 ART treated and 9 untreated) were as follows (median, IQR): age 46 (42-52); time since HIV diagnosis 15 years (6-20); nadir CD4^+^ T lymphocyte count 220 cells/mm^3^ (58-511); plasma HIV-1 RNA 13,040 copies/mL (4,890-22,524); and current CD4^+^ T lymphocyte count 291 cells/mm^3^ (242-555). Between viremic and aviremic participants, the only significant (*P* < 0.05) difference in clinical and demographic factors was for nadir CD4+ T lymphocyte count, which was higher in the viremic group. No demographic or clinical characteristics were available on HIV-uninfected participants.

**Table 1:** Clinical characteristics of studied population.

	N = 39
Male sex	92 (36)
Age (years)	48 (45-55)
White race	59 (23)
Time from HIV diagnosis (years)	17(15-21)
HBV* co-infection	7(3)
HCV** co-infection	26 (10)
Nadir CD4^+^ T lymphocyte count (cells/mm^3^)	66(18-108)
pVL (copies/ml)	<40
Current CD4^+^ T lymphocyte count (cells/mm^3^)	277(177-483)
Current CD4^+^ T lymphocyte percentage	17(14-26)
Current CD4^+^:CD8^+^ T lymphocyte ratio	0.4 (0.2-0.5)
AST (UI/L)	32.5 (26.0-45.0)
ALT (UI/L)	36.5 (24.0-54.0)
BMI (kg/m^2^)	25.7 (23.7-27.6)
PI-containing ART regimen	64 (25)

Data are expressed in % (number) of subjects or median value (interquartile range, IQR).

* Hepatitis B virus (HBV) co-infection defined by positive HBV surface antigen

**Hepatitis C virus (HCV) co-infection defined by positive HCV RNA

AST: aspartate aminotransferase; ALT: alanine transaminase; PI: protease inhibitor

### Collagen deposition and LA CD4^+^ T lymphocyte populations

**Figure 1.**
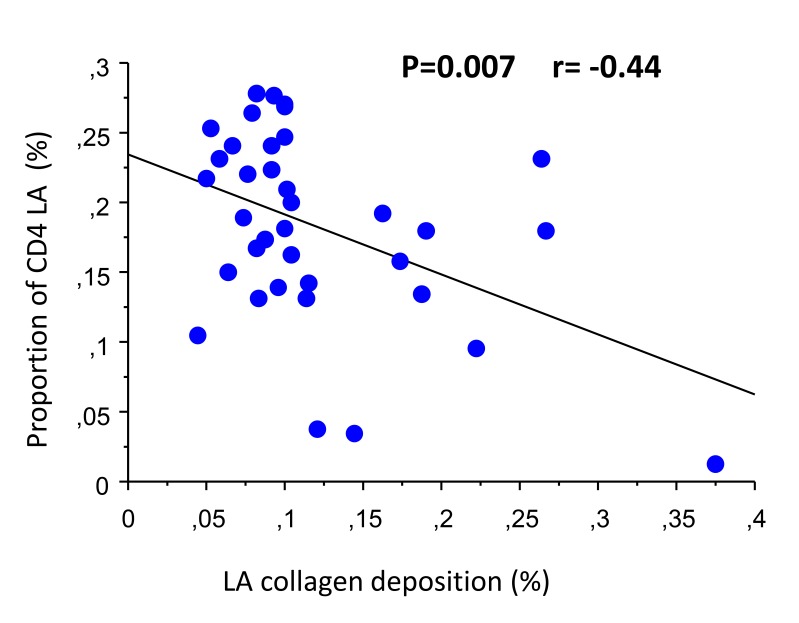
Lymphoid aggregate (LA) fibrosis and CD4+ T lymphocyte frequency. Figure legends:
X axis: percentage of lymphoid aggregate collagen deposition (%)Y axis: proportion of CD4 lymphoid aggregate (%)

Collagen was identified in all GALT biopsy specimens. Median percent LA collagen deposition was 10% (8-13). Proportion of LA CD4^+^ T lymphocytes was 19% (14-24), and frequency significantly negatively correlated with percent LA collagen deposition (r = −0.44, *P* = 0.007 simple regression; r = −0.39, *P* = 0.02, Spearman) (Figure 1). No significant correlations were found between collagen deposition and circulating biomarkers of immune status.

### Intestinal collagen deposition and circulating fibrosis biomarkers

**Figure 2.**
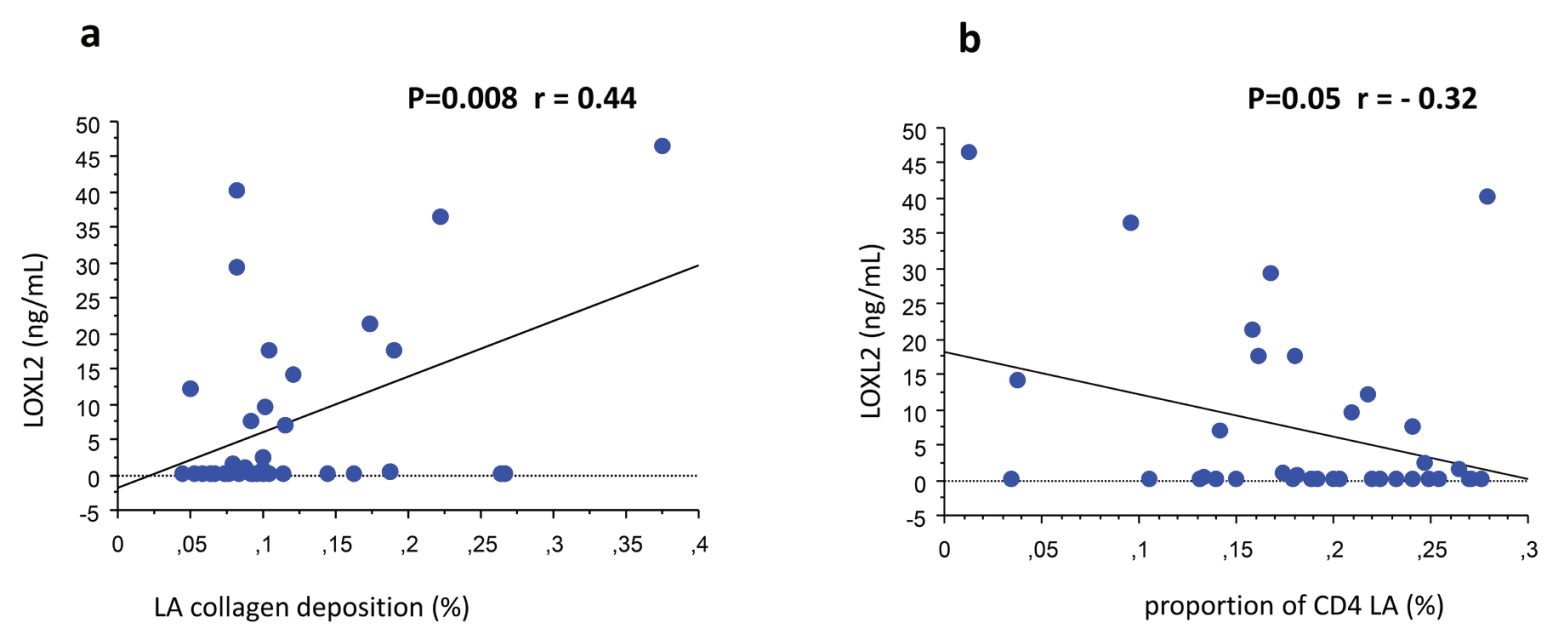
Circulating LOXL2 level correlates with (a) lymphoid aggregate (LA) collagen deposition and (b) CD4+ lymphoid agregate. Figure legends 2a:
X axis: percentage of lymphoid aggregate collagen deposition (%)Y axis: level of LOXL2 (ng/mL) Figure legends 2b:
X axis: proportion of CD4 lymphoid aggregate (%)Y axis: level of LOXL2 (ng/mL)

Levels of circulating fibrosis biomarkers are summarized in Table 2. LOXL2 levels demonstrated a significant relationship with collagen deposition and LA CD4^+^ T lymphocyte counts. Specifically, LOXL2 positively correlated with LA collagen deposition (r = 0.44, *P* = 0.008, simple regression; r = 0.33, *P* = 0.09, Spearman) (Figure 2(a)), and negatively correlated with LA CD4^+^ T lymphocyte counts (r = −0.32, *P* = 0.05, simple regression; r = −0.11, *P* = 0.20 Spearman) (Figure 2(b)).

**Table 2:** Circulating fibrosis biomarker levels among HIV+ aviremic individuals and by immune reconstitution status.*

	Overall (N = 39)	GROUP 1: CD4+ T lymphocyte count < 350 cells/mm^3^ (N = 27)	GROUP 2: CD4^+^ T lymphocyte count ≥350 cells/mm^3^ (N=12)	*P*-value**
TGF-ß_1_ (pg/mL)	12,699 (6,421-17,930)	15,336 (5,661-20,719)	12,316(9,787-14,038)	0.83
TGF-ß_2_(pg/mL)	996(839-1,124)	993 (709-1,085)	1,099(937-1,146)	0.11
TGF-ß_3_(pg/mL)	300(150-404)	255 (99-365)	351 (215-410)	0.17
MMP-2 (pg/mL)	151,129 (124,433-203,864)	179,776 (134,654-258,128)	121,898(105,718-143,495)	0.004
MMP-2 TIMP-1 ratio	4.2 (3.5-6.8)	4.7 (3.5-6.8)	3.2 (2.3-4.7)	0.04
MMP-9 (pg/mL)	7,823 (5,034-12,473)	6,878(4,814-11,054)	7,984(7,081-16,910)	0.20
MMP-9 TIMP-1 ratio	0.20(0.13-0.40)	0.19(0.11-0.37)	0.33 (0.16-0.41)	0.25
TIMP-1 (pg/mL)	42,589 (37,567-47,792)	42,340 (37,567-47,912)	42,780(38,850-45,310)	0.95
CHI-3L1 (pg/mL)	42,900 (26,144-66,183)	39,939 (24,215-64,226)	50,330 (28,614-75,949)	0.42
HA (ng/mL)	47.0 (35.0-86.6)	48.5 (28.3-88.6)	46.0 (36.9-82.6)	0.86
LOXL2 (ng/mL)	0.2(0.2-11.6)	0.2(0.2-11.0)	2.1 (0.2-13.7)	0.16
CICP (ng/mL)	105.6 (84.6-148.6)	110.5(90.4-159.6)	98.1 (62.2-146.3)	0.39
CIC C1Q (μg Eq/mL)	147.4 (95.7-199.9)	128.9(95.7-193.0)	177.2(91.4-233.1)	0.63
PAI-1 (pg/mL)	32,239 (17,623-45,084)	32,239 (13,741-47,196)	30,706 (25,244- 40,171)	0.93
CXCL4 (pg/mL)	1,561,900	1,232,500	2,347,450	0.12
	(401,723-2,845,850)	(250,399-2,734,050)	(1.691,050-2,790,800)	

*Values are expressed as median (interquartile range, IQR)

**P value for Mann-Whitney test comparing group 1 vs group 2

TGF = transforming growth factor, MMP = matrix metalloproteinase, TIMP = tissue inhibitor of MMP, CHI-3L1 = chitinase-3-like protein 1, HA = hyaluronic acid, LOXL2 = lysyl oxidase-like 2, CICP = type I C-terminal collagen pro-peptide, CIC C1Q = circulating immune complexes of complement 1q, PAI = plasminogen activator inhibitor, CXCL4 = CXC chemokine ligand 4

Biomarker level comparisons between HIV+ aviremic, HIV+ viremic and HIV- participants

Fibrosis biomarkers were also compared between viremic and aviremic participants (Table 3). Results showed significantly higher TGF-ß_3_, CIC C1Q, and TIMP-1 levels among HIV+ viremic individuals, with a trend for lower LOXL2 level (0.2 ng/mL [0.2-0.2] vs 0.2 ng/mL [0.2-11.6], *P* = 0.08). When comparing HIV+ aviremic to HIV-individuals, TGF-ß_2_, matrix metalloproteinase-2, TIMP-1, Chi3L1, hyaluronic acid, CICP, and CIC C1Q were significantly lower (*P* < 0.05) in HIV- individuals, while CXCL4 levels were higher in HIV- individuals (Table 3).

In additional exploratory analyses, no significant differences in fibrosis biomarker levels were found by success or failure of circulating CD4^+^ T lymphocyte recovery, with the exception of significantly higher MMP-2 levels and MMP-2:TIMP-1 ratios among HIV+, aviremic individuals with CD4^+^ T lymphocyte counts < 350 cells/mm^3^ compared to those with > 350 cells/mm^3^ (Table 2). Finally, longer duration of HIV infection correlated with higher TIMP-1 levels (r = 0.35, *P* = 0.03), and lower CXCL4 and CIC C1Q levels (simple regression r = −0.35, *P* = 0.03; r = −0.32, *P* = 0.04, respectively).

#### Biomarker level comparisons between HIV+ aviremic, HIV+ viremic and HIV- participants

Fibrosis biomarkers were also compared between viremic and aviremic participants (Table 3). Results showed significantly higher TGF-ß_3_, CIC C1Q, and TIMP-1 levels among HIV+ viremic individuals, with a trend for lower LOXL2 level (0.2 ng/mL [0.2-0.2] vs 0.2 ng/mL [0.2-11.6], *P* = 0.08). When comparing HIV+ aviremic to HIV−individuals, TGF-ß_2_, matrix metalloproteinase-2, TIMP-1, Chi3L1, hyaluronic acid, CICP, and CIC C1Q were significantly lower (*P* < 0.05) in HIV− individuals, while CXCL4 levels were higher in HIV− individuals (Table 3).

**Table 3:** Circulating fibrosis biomarker levels overall among HIV infected and HIV uninfected individuals*

	HIV+ AVIREMIC group (HIV-1 pVL < 50cp/mL) (N = 39)	HIV+ VIREMIC group (HIV-1 pVL > 50cp/ mL) (N=18)	P **	HIV negative group (N = 36)	P ***
TGF-ß_1_ (pg/mL)	12,699(6,421-17,930)	14,890 (10,792-17,696)	0.44	9,746(7,104-16,880)	0.34
TGF-ß_2_(pg/mL)	996(839-1,124)	1,084(963-1,135)	0.19	836(729-946)	**0.005**
TGF-ß_3_(pg/mL)	300(150-404)	377(331-418)	**0.03**	252(151-467)	0.87
MMP-2 (pg/mL)	151,129 (124,433-203,864)	149,560 (120,202-178,693)	0.49	60,028 (60,028-76,312)	**< 0.0001**
MMP-2 TIMP-1 ratio	4.2 (3.5-6.8)	3.0 (2.4-4.2)	**0.04**	3.2 (2.7-4.9)	0.09
MMP-9 (pg/mL)	7,823 (5,034-12,473)	6,232 (4,640-8,936)	0.11	9,108(4,006-16,544)	0.89
MMP-9 TIMP-1 ratio	0.2(0.13-0.40)	0.1 (0.1-0.2)	**0.02**	0.46(0.15-1.1)	**0.02**
TIMP-1 (pg/mL)	42,589 (37,567-47,792)	47,947 (44,130-50,164)	**0.03**	19,099 (16,714-21,418)	**< 0.0001**
CHI-3L1 (pg/mL)	42,900 (26,144-66,183)	39,379(20,117-71,586)	0.62	17,100(12,231-27,442)	**< 0.0001**
HA (ng/mL)	47.0 (35.0-86.6)	60.1 (40.1-67.4)	0.77	32.4 (21.2-49.8)	**0.01**
LOXL2 (ng/mL)	0.2(0.2-11.6)	0.2 (0.2-0.2)	0.08	4.9 (0.2-23.2)	0.15
CICP (ng/mL)	105.6(84.6-148.6)	105.1 (85.2-116.6)	0.66	72 (56-92)	**< 0.0001**
CIC C1Q (μg Eq/mL)	147.4(95.7-199.9)	234(125-350)	**0.01**	5.2 (3.4-7.8)	**< 0.0001**
PAI-1 (pg/mL)	32,239 (17,623-45,084)	35,319(23,692-59,571)	0.22	26,952(16,815-72,171)	0.98
CXCL4 (pg/mL)	1,561,900	2,400,950	0.10	3,296,800(2,312,900-	**< 0.0001**
	(401,723-2,845,850)	(876,244-3,735,200)		5,337,600)	

*Values are expressed as median (interquartile range, IQR)

**P value for Mann-Whitney test comparing HIV+ aviremic group vs HIV+ viremic group

***P value for Mann-Whitney test comparing HIV+ aviremic group vs HIV negative group

pVL = plasma viral load, TGF = transforming growth factor, MMP = matrix metalloproteinase, TIMP = tissue inhibitor of MMP, CHI-3L1 = chitinase-3-like protein 1, HA = hyaluronic acid, LOXL2 = lysyl oxidase-like 2, CICP = type I C-terminal collagen pro-peptide, CIC C1Q = circulating immune complexes of complement 1q, PAI = plasminogen activator inhibitor, CXCL4 = CXC chemokine ligand 4

## DISCUSSION

In this group of HIV+ aviremic individuals on suppressive ART, GALT fibrosis was universally present, with more fibrosis associated with lower CD4^+^ T lymphocyte percentage in LA. Additionally, we found significant correlations among GALT collagen deposition, LA CD4^+^ T lymphocyte frequency, and circulating LOXL2 levels.

HIV+ individuals may display an incomplete immune restoration despite control of HIV replication. Interestingly, all participants had a long history of HIV infection and our sample was enriched for those who persistently had CD4^+^ T lymphocyte counts < 350 cells/mm^3^ (69%). This population can therefore be considered to consist primarily of immunologic non-responders (median CD4^+^ T lymphocyte count 277 cells/mm^3^), and the lack of peripheral immune restoration may partially result from alterations of intestinal immunity. In the first weeks of HIV infection, gut mucosal CD4^+^ T lymphocyte depletion occurs and, with disruption of the mucosal barrier, permits increased microbial translocation. Over time, this leads to chronic stimulation of the adaptive and innate immune systems, perpetuating gut damage, local HIV replication, and CD4^+^

T lymphocyte depletion, culminating in fibrosis [14, [15]. Simultaneously, the lymphatic tissue is affected by structural remodeling, from hyperplasia to follicular involution [16, 17] and/or fibrosis [13]. In this virologically suppressed population, we reported a high level of collagen deposition in all biopsy samples and a negative correlation between LA CD4^+^ T lymphocyte frequency and LA collagen deposition, consistent with previously described results from the complete SCOPE biopsy population [13]. These findings support GALT fibrosis as a contributor to incomplete CD4^+^ T lymphocyte cell restoration in chronic HIV infection despite suppressive ART [13, 14].

This study is the first to explore the relationship between gut collagen deposition and circulating fibrosis biomarkers among HIV+ individuals on suppressive ART. Interestingly, we found that LOXL2 levels increase with LA collagen deposition and are inversely correlated with CD4^+^ T lymphocyte frequency in LA. LOXL2 is a metalloenzyme that plays an important role in the progression of fibrotic disease by promoting collagen and elastin cross-linkage, which is essential for the tensile strength of the ECM [18]. Interestingly, while LOXL2 plays an essential role in angiogenesis, wound healing, and scar formation, [19, 20] it is not thought to be over-expressed in normal hosts [9, 21] and its upregulation has been associated with the development of fibrotic, hematologic, and malignant diseases [9, 10, 19, 21–25].

Thus, our findings suggest a dynamic GALT remodeling process that may prevent complete immune restoration on ART. This hypothesis is supported by the fact that we also observed a strong positive relationship between duration of HIV infection and TIMP-1 levels, suggesting chronic and potentially escalating tissue remodeling [26]. We also observed significantly higher MMP-2 levels and MMP-2:TIMP-1 ratios in HIV+ individuals with CD4^+^ T lymphocyte counts < 350 cells/mm^3^, indicative of ongoing ECM remodeling [27]. While high MMP-2 and TIMP-1 levels have been associated with severity of liver fibrosis, [28, 29] in this cohort levels did not differ by viral hepatitis co-infection status (data not shown).While we did not observe an association between circulating LOXL2 levels and circulating CD4^+^ T lymphocyte counts, the circulating CD4^+^ cells pool is derived from a number of tissues that may not be affected/equally affected by fibrosis, preventing a strong correlation.

In an exploratory subanalysis, comparison of fibrosis biomarker levels between aviremic and viremic HIV+ individuals demonstrated a trend toward lower LOXL2 levels in the viremic group. This result could be explained by the significantly higher nadir CD4^+^ T lymphocyte values among viremic HIV+ individuals, reflecting less immunologic damage and, potentially, lower inflammation and fibrosis in this group. Additionally, most fibrosis biomarkers were significantly lower in HIV− group compared to the HIV+ aviremic group, as expected. While CXCL4 levels were higher among HIV− persons, this was also expected: we previously demonstrated among Multicenter AIDS Cohort participants that CXCL4 levels are lower among persons with failure to immune reconstitute on ART, [30] and *in vitro* data suggest that lower CXCL4 levels in HIV+ persons may be a marker of successful immune evasion by the virus [31]. Thus, persons with lower CXCL4 levels on suppressive ART may be reflective of greater immunologic devastation. Additionally, CXCL4 originates from activated platelets, and circulating levels are elevated in atherosclerosis and cardiovascular disease, [32, 33] malignancy, [34–36] and systemic sclerosis [12, 37]. Thus, many factors affect CXCL4 levels.

Finally, no significant difference in LOXL2 levels was observed between HIV− and HIV+ aviremic participants, which is not surprising as, given that LOXL2 upregulation is associated with pathologic states, levels are low in most persons. Notably, the HIV− group was a sample of stored plasma from unmatched HIV− individuals with unknown medical histories and no GALT biopsy data, so this finding must be interpreted with caution. Additionally, circulating LOXL2 levels have not been measured in many clinical cohorts, so the effect of factors such as age, sex, obesity, cardiovascular disease, and liver disease on LOXL2 levels are unknown. Thus, we believe that the lack of statistical difference in levels between HIV+ aviremic persons and unmatched HIV− persons does not take away from the strong relationship observe between circulating LOXL2 levels and the extent of GALT fibrosis and CD4^+^ T lymphocyte depletion observed in HIV+ aviremic adults.

Our study has several limitations: The cross-sectional design and small sample size may have prevented us from observing additional statistically significant relationships between other circulating fibrosis biomarkers and GALT collagen deposition, particularly for markers that may fluctuate more widely within individuals and/or have higher coefficients of variation. Additionally, we were not powered to look at gender differences in the observed relationships, as the vast majority of participants with available samples were males. The majority of our participants also identified as being of white race. Thus, our results cannot be generalized to women and/or the global HIV+ population. Finally, HIV− control samples came from an unmatched convenience sample of persons without clinical profiling.

In conclusion, LOXL2 levels increase with GALT lymphoid aggregate collagen deposition and are inversely correlated with lymphoid aggregate CD4^+^ T lymphocyte frequency in HIV+ adults on suppressive ART. Further studies are needed to assess the utility of circulating LOXL2 as a noninvasive marker of fibrotic tissue burden in treated HIV infection. Finally, specific inhibitors of LOXL2 should be explored for the prevention and treatment of fibrotic disease in chronic HIV infection.
